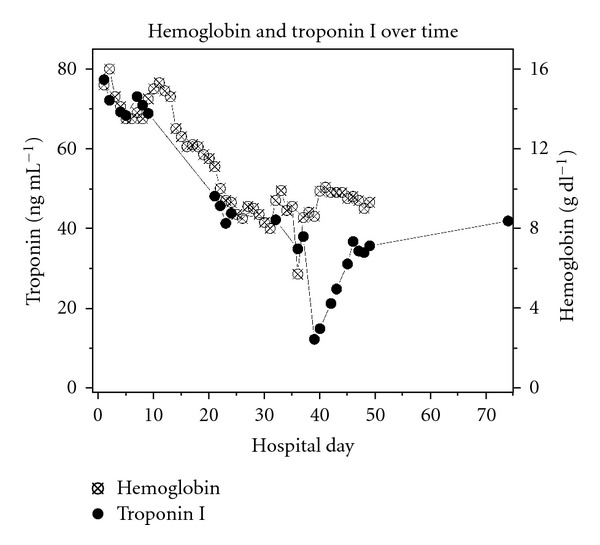# Fluctuation of Spuriously Elevated Troponin I: A Case Report

**DOI:** 10.1155/2012/585879

**Published:** 2012-03-25

**Authors:** Sam Ghali, Keith Lewis, Viviane Kazan, Neezam Altorok, Jamil Taji, Mohammad Taleb, Kiranmayee Lanka, Ragheb Assaly

**Affiliations:** The University of Toledo Medical Center, Toledo, OH 43614, USA

## Abstract

Serum troponin is a useful laboratory study for the diagnosis of acute myocardial infarction. However, elevations can also be seen in a variety of other diseases processes. Falsely positive troponin values caused by interference with current troponin assays have been reported. We report a unique case that demonstrates the fluctuation of falsely elevated troponin correlating with hemoglobin, serving as a marker of heterophile antibody levels. A 74-year-old gentleman presented to our Emergency Department with a several-day history of increasing shortness of breath associated with a new-onset chest pain and a troponin I level of 77.28 ng/mL. Throughout his stay, fluctuations in measured troponin levels correlated strongly with fluctuations in hemoglobin levels. Several investigations confirmed false elevated troponin levels secondary to heterophile antibody interference. We conclude that hemoglobin trending in our patient represented a surrogate measure of his heterophile antibody titers with time and that fluctuations in these levels correlated with respective fluctuations in the falsely elevated troponin levels.

## 1. Introduction

 Cardiac troponins T (cTnT) and I (cTnI) are regulatory proteins that control the calcium-mediated interaction between actin and myosin [1]. The skeletal and cardiac isoforms of cTnT and cTnI are distinct, and skeletal isoforms are not detected by the monoclonal antibody-based assays currently in use [2]. This specificity for cardiac isoforms is the basis for the clinical utility of cTnT and cTnI assays.

 Relying on history, physical examination, and ECG abnormalities to diagnose acute myocardial infarction may often lead the clinician astray. Thus, the diagnosis of an acute myocardial infarction has become increasingly dependent upon the evaluation of cardiac enzymes, particularly cardiac troponins [2, 3].

 In addition to acute myocardial infarction, elevated serum troponins can also be seen in a variety of other diseases including sepsis or critical illness, tachycardia, LVH, heart failure, pulmonary embolism, myocarditis, myocardial trauma, and renal failure [4, 5]. Although irreversible myocyte damage is the usual presumed mechanism responsible for troponin elevation, several additional mechanisms are believed to be responsible for elevated serum troponins in the aforementioned pathological states, including endothelial dysfunction, loss of membrane integrity with leakage of the free cytosolic troponin pool, stretch-mediated troponin release, and impaired renal excretion [6].

 Falsely elevated troponin values caused by interference with current troponin assays have been reported. We report a unique case that demonstrates the fluctuation of falsely elevated troponin correlating with hemoglobin, serving as a marker of heterophile antibody levels [7–17].

## 2. Case Presentation

 A 74-year-old man, a retired railroad conductor, with a history of hypertension, hyperlipidemia, and noninsulin-dependent diabetes mellitus presented to our Emergency Department with a several-day history of increasing shortness of breath associated with a new-onset chest pain. ECG performed in the Emergency Department showed a right bundle branch block, left ventricular hypertrophy, and left atrial enlargement. Troponin I was elevated at 77.28 ng/mL. (Beckman-Coulter's Access AccuTnl Assay; reference range 0.00–0.04) The remainder of the cardiac enzymes were essentially normal: myoglobin (50 ng/mL), CK-MB (5.2 ng/mL), and creatine kinase (74 IU/L). D-Dimer was normal at 0.33 mcg/mL. BUN was 13 mg/dL and creatinine was normal at 0.65 mg/dL (reference range 0.64–1.27). Standard Acute Coronary Syndrome protocol was initiated, and the patient was admitted to the hospital. Transthoracic echocardiography showed left ventricular hypertrophy, mild diastolic dysfunction, and a normal ejection fraction with no evidence of wall motion abnormality. Troponin levels remained elevated throughout the entire hospitalization, in continued disproportion to the other cardiac enzymes, (Myoglobin, CK-MB, and CK) which remained either normal or very mildly elevated throughout.

 His hospital course was complicated by bibasilar pneumonia and atelectasis requiring multiple bronchoscopies, intubation with ventilatory support, and ultimately a tracheostomy; bilateral lower extremity deep venous thromboses, for which he underwent successful inferior vena cava filter placement; a progressive thrombocytopenia which proved to be Heparin-Induced Thrombocytopenia-antibody positive; and finally, a life-threatening bleeding duodenal ulcer.

 A complete blood count trend analysis revealed a significant two-week down-trending of the patient's hemoglobin and hematocrit values; retrospectively a result of the slowly bleeding ulcer. Troponin levels was obtained serially throughout.  Figure 1 is a graph showing all the patient's troponin I levels recorded at our hospital, with a juxtaposed graphing of his hemoglobin levels.

 As the duodenal ulcer proved to be incendiary, and refractory to temporizing measures, the patient ultimately developed hemorrhagic shock and was taken emergently to the operating room where he underwent an exploratory laparotomy with pyloroplasty and successful suture ligation of the ulcer.

 In addition to extensive resuscitation with normal saline, the patient had required a total of 18 units of PRBC. 12 of these units were given within 24 hrs. Intraoperatively, the patient received 5 units of PRBC, 10 units of FFP, and 5 units of platelets.

## 3. Investigations

 A patient's blood sample was sent out for a troponin I level on two separate occasions to a near-by hospital laboratory that used the Siemens ADVIA Centaur Immunoassay as opposed to the Beckman-Coulter Access AccuTnl Assay used by our hospital. Both times the result returned at <0.01 ng/mL. Additionally, a random troponin T level measured by Associated Regional and University Pathologists Laboratories using the Electrochemiluminescent Immunoassay was only 0.13 ng/mL.

 A heterophile antibody by latex agglutination was also sent out to ARUP Laboratories and returned negative. However, since this screen only tests for those heterophile antibodies known to develop in response to infection with Epstein-Barr Virus, this test was of limited value. Rheumatoid factor, which also may induce heterophilic antibodies [18], was also negative in our patient.

 We contacted Beckman-Coulter and request a formal investigation for Heterophile antibodies for our patient. The following blocking reagents, produced by Scantibodies Laboratory, were introduced into the patient's serum and again run through the immunoassay: Goat IgG, Mouse IgG, Rabbit IgG, Bovine IgG, Poly Mak 33, Scavenger ALP, AP Mutein, HBR-1, HBR-Non Murine, and TRU Block [19].

 A blocking reagent is a preparation which, when added to immunoassay reagents, has the ability to block the binding of heterophile antibodies, effectively preventing heterophilic interference with another measured value. This is manifested by normalization or near-normalization of the measured value (in this case troponin I) after introduction of the blocking reagent. A blocking reagent can be either a specific blocking reagent, meaning that it has the ability to block only one specific heterophile antibody, or a nonspecific blocking reagent, meaning that it is capable of block heterophile antibodies in general, “nonspecifically” [19].

 The first 7 reagents listed previously are specific heterophile antibody blocking reagents. The last 3 are nonspecific heterophile antibody blocking reagents. 9 out of the 10 blockers had no effect on the resulting troponin I level. However, the addition of HBR-1, a nonspecific blocking reagent, reduced the troponin result by greater than 90% of the original value, indicating successful blockage of our patient's heterophile antibodies, drastically diminishing the spurious elevation, with near-normalization of the level.

## 4. Discussion

 We suspected heterophile antibody interference in our patient for several reasons. The troponin elevation could not be explained by acute myocardial damage. There was no evidence of wall motion abnormalities, there was no clinical suggestion of myocarditis, the degree of LVH was moderate at best, and the degree of troponin elevation was inappropriate and inconsistent with what would be expected for any of these pathologies. Also, the troponin elevations were in extreme disproportion to the remainder of the cardiac enzymes, and the typical pattern of decay known of the troponin was not demonstrated. Finally, the presence of immune interference was suspected due to the dramatic fluctuations in troponin levels correlating with blood loss, blood transfusions (replacements), and overall volume status.

 The term “heterophile antibodies” has historically been used in reference to specific heterophile antibodies, produced by the human immune system in response to Epstein-Barr virus infection. These specific heterophile antibodies react to antigens from phylogenetically unrelated species. They agglutinate sheep erythrocytes, used in the classic Paul-Bunnell test, horse erythrocytes, used in the “Monospot” test, as well as ox and goat erythrocytes [20].

 Probably a better term, antianimal antibodies, encompasses the many different human heterophile antibodies that have the ability to bind to immunoglobulins of many different animal species. It is estimated that 10–40% of humans possess antianimal antibodies. “Anti-animal antibodies (IgG, IgA, IgM, IgE class, anti-isotype, and anti-idiotype specificity) arise as a result of iatrogenic and noniatrogenic causes and include human anti-mouse, -rabbit, -goat, -sheep, -cow, -pig, -rat, and -horse antibodies and antibodies with mixed specificity. Circulating antibodies can reach gram per liter concentrations and may persist for years” [20].

 Reagents used in contemporary troponin immunoassays are derived from immunoglobulins of other species. These antianimal antibodies, or “heterophile antibodies”, have the capability to interfere with the assays by simply binding these immunoglobulins, resulting in spurious troponin elevations. Even the newer ultrasensitive three-site sandwich troponin I immunoassays have been found to produce similar false positive results [21].

Figure 1 demonstrates two key drops in the measured troponin values. The first drop was from 68.83 ng/mL to 47.11 ng/mL (a net of 221221.72 ng/mL) and occurred over a period of 13 days. The patient was transfused 6 units of PRBC's during this time period. No additional troponins were drawn during this 13-day period. The hemoglobin levels essentially mirrored the steady drop in troponin. The continued loss and dilution of the patient's blood steadily overcame the rate of heterophile antibody production, effectively mitigating the interference with the troponin assay. This manifested as a consistent, gradual decline in measured troponin levels observed on the graph.

 The second troponin drop produced a glaring, abrupt dip on the troponin curve, as this drop was from 35.23 ng/mL to 11.20 ng/mL (a net of −24.03 ng/mL) over a period of only less than 32 hours. This highly precipitous decrease in troponin was the result of the patient's extensive upper gastrointestinal bleeding, copious fluid resuscitation, and massive blood transfusions described previously, which served to purge and dilute his heterophile antibodies, again mitigating interference with the troponin assay. Furthermore, according to our theory it follows that his troponin levels should gradually but steadily climb back up, once given an opportunity for his antibody levels to build back up to his baseline. Looking at the graph we can see that this trend too was demonstrated.

Since the in vitro addition of HBR-1 to the patient's serum reduced the troponin result by greater than 90% of the original value, we know that the patient does indeed posses heterophile antibodies that interfere with the Beckman-Coulter Access AccuTnl Assay. Since HBR-1 is a nonspecific heterophile antibody blocker, it remains unknown what the specific heterophile antibody(ies) are.

 Several sources have been implicated as possible causes for inducing heterophile antibodies in humans, including exposure to animals, special diets, deliberate immunization, rheumatoid factors, blood transfusions, autoimmune diseases, dialysis, certain medications, and cardiac myopathy [19]. The elicitation of heterophile antibodies in our patient may have been the result of one or several of these causative factors.

 We conclude that hemoglobin trending in our patient represented a surrogate measure of his heterophile antibody titers with time and that fluctuations in these levels correlated very strongly with respective fluctuations in the falsely elevated troponin levels.

## Figures and Tables

**Figure 1:**